# The ubiquitin-proteasome system in circadian regulation

**DOI:** 10.3389/fnins.2025.1632905

**Published:** 2025-08-26

**Authors:** Kara M. Costanzo, Matthew V. Prifti, Sokol V. Todi, Ryan D. Mohan

**Affiliations:** ^1^Department of Pharmacology, Wayne State University School of Medicine, Detroit, MI, United States; ^2^Department of Neurology, Wayne State University School of Medicine, Detroit, MI, United States

**Keywords:** ubiquitination, deubiquitination, transcriptional regulation, molecular clock, protein homeostasis

## Abstract

To align sleep–wake behavior and internal physiology with the Earth’s 24-h light–dark cycle, organisms rely on circadian clocks–endogenous timekeeping systems that anticipate and adapt to daily environmental changes. These clocks are governed by transcription-translation feedback loops that produce rhythmic oscillations in gene expression, including key regulators such as PERIOD and CRYPTOCHROME. The timing and stability of these proteins are tightly controlled by post-translational mechanisms, including ubiquitin-mediated degradation. The ubiquitin-proteasome system (UPS) ensures that clock proteins are cleared at precise times within the circadian cycle, a process which is essential for resetting the molecular clock and sustaining robust circadian rhythms. Disruption of this process can have profound impacts on human health and contribute to impairments in sleep timing, circadian phase, and rhythm amplitude. In this review, we focus on the mechanistic role of the UPS in circadian clock regulation, summarize key E3 ligases and deubiquitinating enzymes implicated in clock protein turnover, and highlight the essential role of the UPS on sleep timing and overall circadian biological homeostasis.

## 1 Introduction

In diverse organisms, circadian clocks regulate the timing of physiological and behavioral processes in alignment with the 24-h day. These clocks consist of cell-autonomous molecular oscillators that synchronize internal time with environmental light–dark cycles (Takahashi, 2017). In mammals, the central pacemaker is in the suprachiasmatic nucleus (SCN), a bilateral structure in the anterior hypothalamus. In rodents, the SCN is composed of approximately 20,000 neurons, each functioning as an autonomous oscillator (Abrahamson and Moore, 2001; Fagiani et al., 2022). In humans, estimates of SCN neuron numbers range from ∼20,000 to over 50,000, depending on age and methodology (Wang et al., 2015; Hofman et al., 1996; Patton and Hastings, 2018). The SCN receives light input directly from the retina and conveys time-of-day signals to peripheral clocks throughout the body, ensuring systemic synchronization (Buijs et al., 2016). The peripheral clocks are found in nearly every tissue examined, including the lungs, liver, heart, and skeletal muscle (Fagiani et al., 2022).

At the molecular level, circadian rhythms are primarily driven by transcriptional-translational feedback loops (TTFLs) and rhythmic posttranslational modifications, such as phosphorylation and ubiquitination (Albrecht, 2012). TTFLs regulate the near-24-h expression of core clock genes via negative feedback (Abdalla et al., 2022), while additional layers of control–including post-transcriptional, translational, and protein degradation pathways–further fine-tune these oscillations (Mendoza-Viveros et al., 2017). As research has progressed, it has become clear that a relatively small set of clock genes generates highly precise timing signals that cascade to influence a broad array of cellular functions (Fuhr et al., 2015).

The circadian regulation of gene expression plays a fundamental role in maintaining physiology. Early studies revealed that up to 20% of the genome is under circadian control (Panda et al., 2002a,Storch et al., 2002; Zhang et al., 2014). More recent work in primates has expanded this view significantly. Analyzing the transcriptome of non-human primates, Mure et al. (2018) found that over 80% of protein-coding genes exhibit daily rhythmic expression across major tissues and brain regions. These genes span a broad array of cellular and biochemical pathways, highlighting circadian regulation as one of the most pervasive systems for coordinating gene expression. Importantly, 82.2% of genes encoding proteins classified as druggable targets by the U.S. Food and Drug Administration also show rhythmic transcription (Mure et al., 2018; Fagiani et al., 2022). To maintain coherence across the body, the circadian system must continuously align with environmental cues and internal signals, coordinating local cellular clocks and integrating tissue-specific rhythms into a unified temporal framework that governs physiology and behavior.

As one of the essential regulatory pathways in circadian biology, the intricate dynamics between the ubiquitin-proteasome system (UPS) and circadian rhythms have emerged as a fundamental axis of biological regulation. Dysfunction of the UPS is linked to altered sleep timing, reduced circadian rhythm amplitude, and impaired phase resetting, highlighting UPS components as promising targets for correcting circadian disruptions and related disorders. Chemical screens have identified small molecules that modulate the ubiquitin-mediated degradation of core clock proteins, offering potential strategies for resetting the circadian clock (Hirota et al., 2012). Additional compounds targeting key regulators have shown efficiency in fine-tuning circadian physiology (Solt et al., 2012; Chen et al., 2018c), highlighting the potential of combining these approaches with time-of-day specific UPS modulation to enhance therapeutic precision and efficacy.

In this review, we examine how the UPS contributes to circadian clock function by regulating the stability and turnover of rhythmically expressed clock proteins. We highlight key E3 ubiquitin ligases and deubiquitinating enzymes (DUBs) that modulate the abundance of core clock components and their downstream effectors and discuss how disruptions in these processes can lead to circadian misalignment and disease.

## 2 Overview of circadian machinery and clock associated ubiquitination

### 2.1 The *Drosophila* circadian clock

Much of the foundational knowledge of circadian biology came from *Drosophila melanogaster*. The first core clock gene, *period* (*per*), was discovered in *Drosophila* mutants with disrupted behavioral rhythms (Konopka and Benzer, 1971). *per* plays a central role in regulating circadian timing by encoding the period protein, which functions as a transcriptional repressor. Taking advantage of high-throughput genetic screening in *Drosophila*, Sehgal et al. (1994) identified *timeless* (*tim*) as the second core clock gene, showing that its protein product forms a complex with per (Hall, 2003; Sawyer et al., 2006; Sehgal et al., 1994). This per-tim complex accumulates in the cytoplasm before entering the nucleus to inhibit its own transcription by repressing the transcriptional activators Clock (Clk) and cycle (cyc) (Crane and Young, 2014; Lee et al., 1999; Ashmore et al., 2003; Saez and Young, 1996). Together, Clk (Allada et al., 1998) and cyc (Rutila et al., 1998) form a heterodimer that activates *per* and *tim* transcription. The resulting per-tim complex feeds back to repress Clk-cyc activity, and their subsequent degradation relieves this repression thus allowing Clk-cyc to initiate a new cycle (Tataroglu and Emery, 2015; Lee et al., 1999; Ukita et al., 2022).

Circadian light entrainment in *Drosophila* is mediated by the light-sensitive photoreceptor cryptochrome (cry) (Busza et al., 2004; Emery et al., 1998). In *Drosophila*, cry responds to blue light by binding to tim and recruiting the E3 ubiquitin ligase jetlag (jet), marking tim for proteasomal degradation and thereby resetting the molecular clock (Stanewsky et al., 1998; Lin et al., 2001; Koh et al., 2006). This light-dependent tim degradation enables the circadian system to synchronize with external light-dark cycles. Importantly, in *Drosophila*, cry serves solely as a photoreceptor and does not participate in transcriptional repression.

**FIGURE 1:**
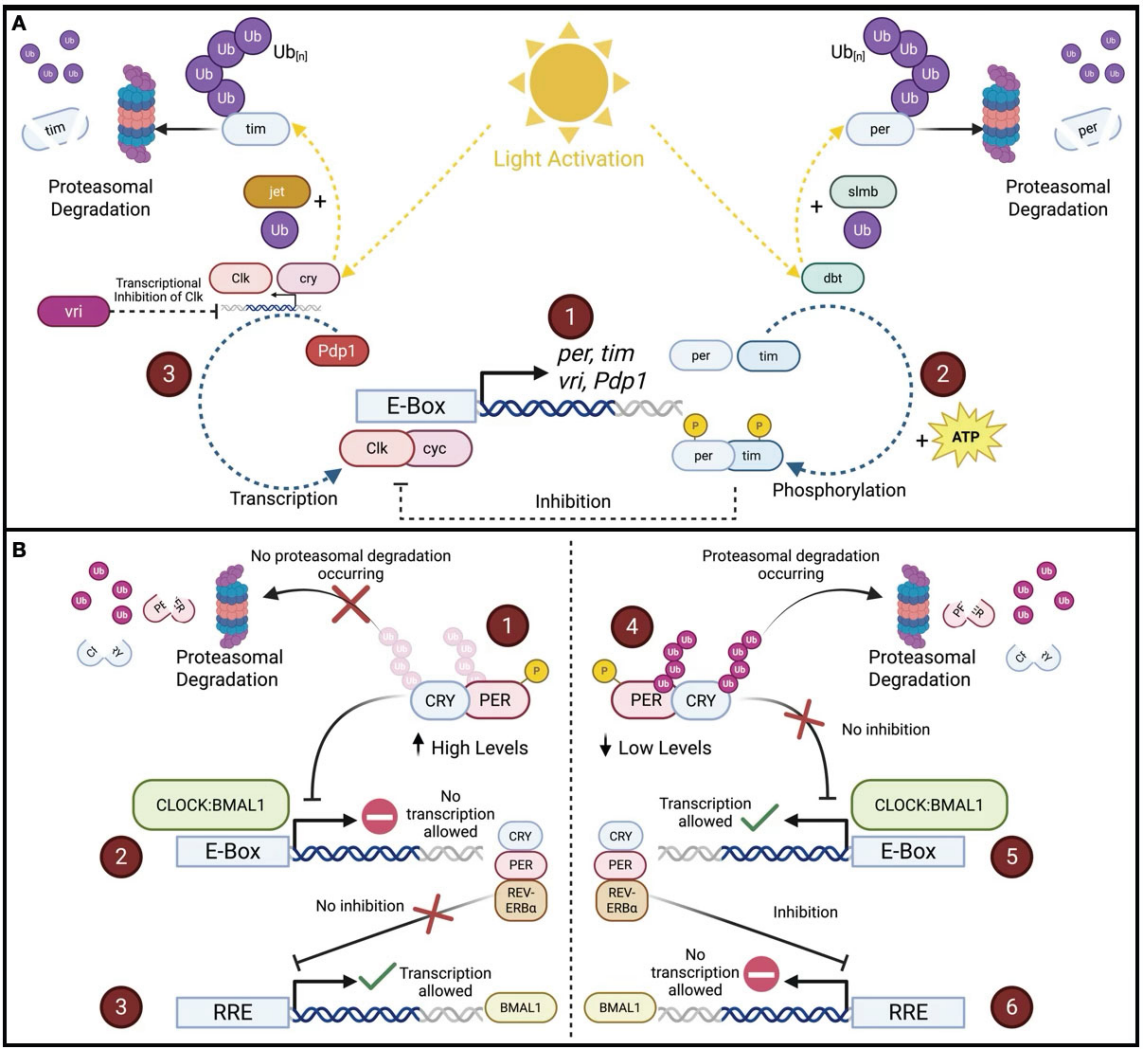
Ubiquitin is a small, evolutionarily conserved protein that serves as a post-translational modifier, governing among many things the stability, localization, and function of a wide range of cellular proteins and processes. Ubiquitination is carried out through a hierarchical cascade involving ATP, E1 activating enzymes, E2 conjugating enzymes, and E3 ligases, which together mediate the covalent attachment of ubiquitin to specific target proteins, typically on lysine residues. The outcome of ubiquitination depends on the nature of the ubiquitin linkage. The most recognized K48-linked chains are generally signals for proteasomal degradation, while K63, K33, K27 and other atypical lysine linkages modulate trafficking, DNA damage responses and other processes. Ubiquitin-dependent mechanisms are essential for cellular homeostasis. **(A)** Illustrative representation of the ubiquitination cascade. This includes activation of ubiquitin by E1 and ATP hydrolysis, transfer of the activated ubiquitin to the E2 conjugating enzyme and substrate-specific ligation of ubiquitin mediated by the E3 ligase resulting in a covalently attached ubiquitin. Shown in the panel is also a DUB (deubiquitinase), representing the class of enzymes that dismantle ubiquity linkages. **(B)** Proteasomal degradation of core circadian rhythm proteins such as PER. Phosphorylated PER is ubiquitinated in an ATP-dependent process involving E1 activating enzymes, E2 conjugating enzymes, and E3 ligases including β-TrCP1/β-TrCP2 and slmb. Once polyubiquitinated, PER can be deubiquitinated by DUBs, such as USP2 and USP14, or targeted for degradation via the AAA-ATPase VCP/p97, which facilitates delivery to the 26S proteasome. At the proteasome, the 19S regulatory particle first recognizes the polyubiquitin chain. The deubiquitinating enzyme RPN11 at the proteasome lid cleaves ubiquitin from the substrate in a tightly coupled, ATP-dependent manner. The substrate is then unfolded and translocated into the 20S core particle, where it is proteolytically degraded into short peptides. The second 19S cap facilitates substrate exit and release of degradation products, completing the proteasomal processing cycle. This regulated degradation of PER in turn plays a key role in maintaining circadian timing and the periodic repression of CLOCK:BMAL1 activity.

Three additional significant circadian components have been identified using *Drosophila* genetic screenings. double-time (dbt; also known as disks overgrown, or dco), shaggy (sgg), and vrille (vri) refine the core circadian feedback loop in flies (Price et al., 1998; Panda et al., 2002b,Blau and Young, 1999). dbt, an ortholog of the mammalian casein kinase Iε (CkIε), phosphorylates per to regulate its stability and nuclear entry timing, creating essential delays in the feedback cycle (Price et al., 1998; Kloss et al., 1998; Suri et al., 2000; Vielhaber et al., 2000). sgg phosphorylates tim, affecting the timing of per/tim nuclear entry, while vri acts as a transcriptional repressor of Clk, contributing to rhythmic gene expression (Panda et al., 2002b,Blau and Young, 1999; Figure 1A).

### 2.2 The mammalian circadian clock

Parallel discoveries in mammals revealed both similarities and distinctions within the circadian machinery. The first mammalian core clock gene, CLOCK, was identified through studies of mice with abnormal circadian behavior (Vitaterna et al., 1994; King et al., 1997). CLOCK is a transcription factor that heterodimerizes with BMAL1–the mammalian ortholog of *Drosophila* cycle–to form the core CLOCK:BMAL1 complex (Allada et al., 1998; Rutila et al., 1998; Lowrey and Takahashi, 2011; Ye et al., 2014). This complex binds to E-box elements in target gene promoters to drive the rhythmic transcription of PER1, PER2, PER3, CRY1, and CRY2 (Shearman et al., 1997; Kume et al., 1999; Mendoza-Viveros et al., 2017).

In mammals, as PER and CRY proteins accumulate, they dimerize, enter the nucleus, and inhibit CLOCK:BMAL1 activity, forming the negative feedback loop that is central to circadian timekeeping (Sehgal et al., 1994; Meyer et al., 2006; Lee et al., 1999). In contrast to *Drosophila*, mammalian CRY proteins (CRY1 and CRY2) do not function as photoreceptors. Instead, they are essential components of the negative feedback arm, acting directly as transcriptional repressors (Griffin et al., 1999; Kume et al., 1999; Patke et al., 2020). In the mammalian circadian clock, CRY proteins essentially replace tim and partner with PER proteins to form repressor complexes that drive rhythmic gene expression (Yagita et al., 2002; Siepka et al., 2007; Brenna et al., 2019; Figure 1B).

Additional feedback loops exist to enhance the stability and precision of circadian oscillations. One key loop involves the orphan nuclear receptor REV-ERBα and *ROR* genes, whose expression is activated by the CLOCK:BMAL1 complex and feeds back to repress *BMAL1* transcription (Preitner et al., 2002; Cho et al., 2012; Stojkovic et al., 2014; Lowrey and Takahashi, 2011). This secondary loop influences the amplitude and phase of clock gene expression. REV-ERBα expression is rhythmic, peaking when PER levels are low, and is disrupted in *CLOCK, PER1/2*, or *CRY1/2* mutant mice–highlighting its dependence on the core feedback loop (Preitner et al., 2002). Importantly, along with other clock proteins, REV-ERBα is subject to ubiquitin-dependent degradation by E3 ubiquitin ligases such as FBXW7 and UBE3A (Gossan et al., 2014; Zhao et al., 2016), as discussed in more detail in Section “4 E3 ligases and deubiquitinating enzymes in circadian regulation.”

### 2.3 Turnover of core clock proteins

The timed degradation of core clock proteins by the ubiquitin-proteasome system ensures proper cycling between repression and activation within the feedback loop, maintaining clock period length. For example, light-induced degradation of tim depends on phosphorylation-triggered ubiquitination (Naidoo et al., 1999), while cry levels modulate tim stability by inhibiting its ubiquitination (Lin et al., 2023). Notably, clock resetting can proceed without tim-cry dissociation, as the UPS targets the complex for degradation (Lin et al., 2023). The timely degradation of these clock proteins is essential to release transcriptional repression and initiate the next circadian cycle. UPS mediated protein turnover thus functions as a molecular timer, governing the stability and turnover of core clock proteins to sustain precise 24-h oscillations. Next, we focus on the role of ubiquitin in circadian regulation.

## 3 Overview of ubiquitination

### 3.1 Ubiquitin

**FIGURE 2:**
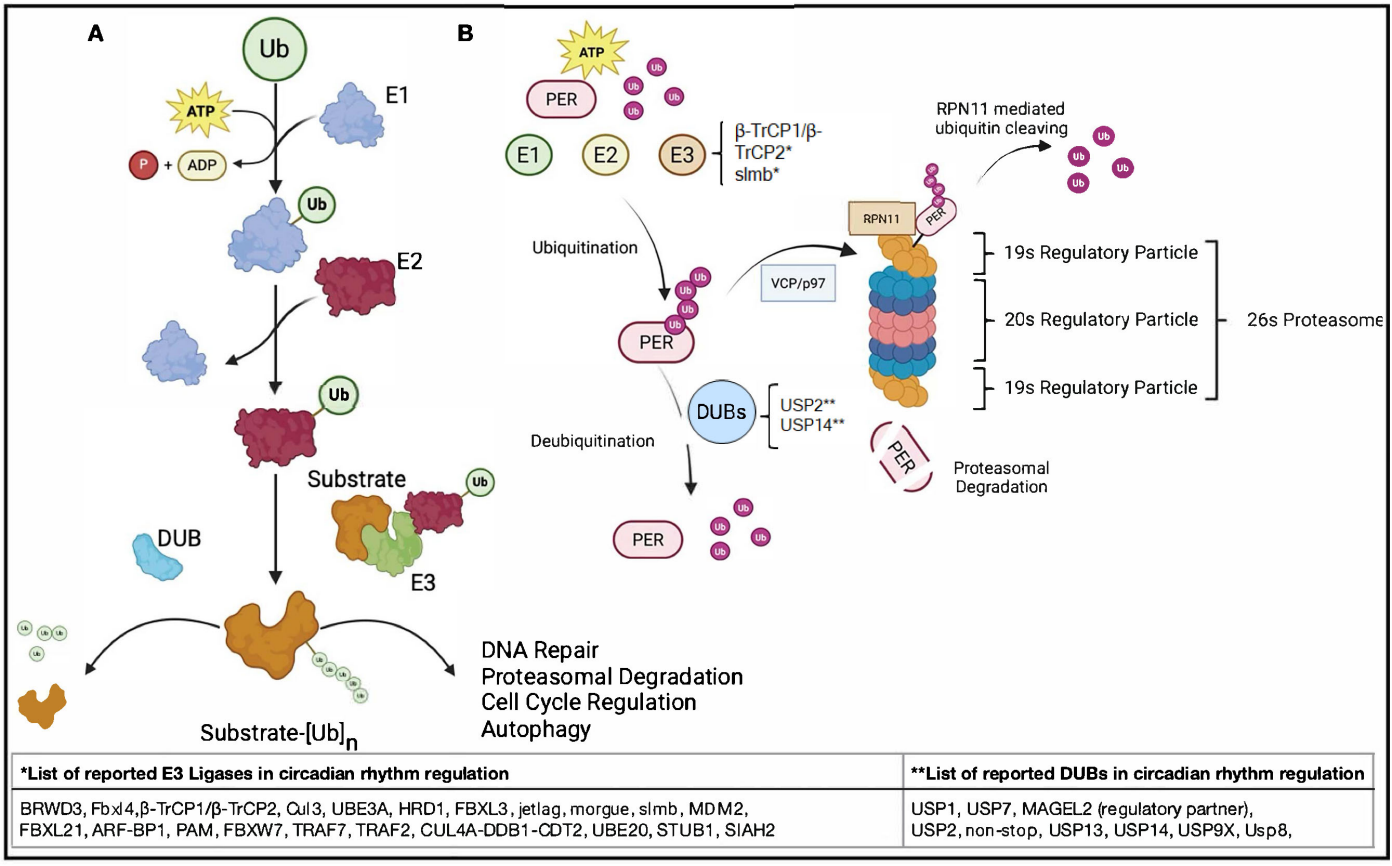
Simplified molecular mechanisms of the UPS in regulation of circadian transcription-translation feedback loops (TTFLs) in *Drosophila* and mammals. **(A)** The core circadian feedback loop and light-dependent regulation in *Drosophila melanogaster*. (1) In the *Drosophila* circadian clock, the transcription factors Clock (Clk) and cycle (cyc) bind to E-box elements in the promoters of key clock genes, including *period (per), timeless (tim), vrille (vri)*, and *PAR domain protein 1*ε *(Pdp1)*, to initiate rhythmic transcription. (2) As per and tim proteins accumulate in the cytoplasm, they are progressively phosphorylated. per is targeted by the kinase doubletime (dbt), and under light conditions, phosphorylated per is recognized by the E3 ligase slmb, tagged with ubiquitin, and sent to the proteasome for degradation. At the same time, light activates the blue-light photoreceptor cry, which binds tim and recruits the F-box protein jetlag (jet) to promote its ubiquitination and degradation. These light-sensitive degradation pathways contribute to the daily resetting of the clock. When per and tim escape degradation and form a stable complex, they translocate to the nucleus and inhibit Clk/cyc activity, closing the negative feedback loop that suppresses further *per* and *tim* transcription. (3) Meanwhile, vri and Pdp1, also regulated by Clk/cyc, feedback to modulate Clk expression. vri acts as a repressor, while Pdp1 promotes transcription, creating a secondary loop that helps sustain rhythmic Clk levels. Together, these tightly coupled feedback systems drive ∼24-h oscillations in gene expression and behavior. **(B)** The core transcriptional-translational feedback loops in the human circadian clock. (1) In the mammalian circadian system, when the phosphorylated PER–CRY complex is stable and not targeted for proteasomal degradation, its levels remain high. (2) This complex enters the nucleus and inhibits CLOCK:BMAL1-driven transcription from E-box elements, reducing the expression of PER, CRY, and REV-ERBα. (3) Because REV-ERBα is not being transcribed, its repression of ROR response elements (RREs) is relieved, allowing BMAL1 transcription to occur. (4) On the other hand, when PER and CRY are phosphorylated, ubiquitinated, and directed to the proteasome for degradation, their levels fall, lifting inhibition of CLOCK:BMAL1 activity. (5) This reactivates E-box–mediated transcription of PER, CRY, and REV-ERBα. (6) As REV-ERBα accumulates, it binds RREs and represses BMAL1 transcription, completing the feedback loop. Together, these interactions create a tightly regulated ∼24-h rhythm in gene expression.

Ubiquitin (Figure 2A) is a small, highly conserved 76-amino acid protein that is covalently attached to target proteins through a three-enzyme cascade: the ubiquitin-activating enzyme (E1), ubiquitin-conjugating enzyme (E2), and ubiquitin ligase (E3) (Ukita et al., 2022; Bachiller et al., 2020; Leestemaker and Ovaa, 2017). This post-translational modification, termed ubiquitination, regulates a wide range of cellular processes, including protein degradation, intracellular trafficking, and signal transduction (Liao et al., 2024). Its critical role is underscored by 96% sequence identity between yeast and humans (Mavor et al., 2016; Ozkaynak et al., 1987). Ubiquitin induces an immense range of changes in cellular protein activity, including alterations in protein conformation, localization, interactions, and additional, subsequent post-translational modifications (Komander and Rape, 2012; Liao et al., 2022).

Beyond protein turnover, ubiquitination plays a crucial role in regulating chromatin dynamics and cellular signaling pathways. Histones are among the most heavily ubiquitinated proteins, and these modifications serve as molecular signals that influence chromatin accessibility and gene expression by recruiting or blocking regulatory factors (Vaughan et al., 2021). Recently, histone ubiquitination has emerged as a key epigenetic mechanism for transcriptional regulation (Shu et al., 2025). More broadly, ubiquitin signaling orchestrates cellular decisions that maintain physiological balance. When this regulation is disrupted, it can lead to pathological outcomes, including neurodegenerative diseases, cancers, and metabolic disorders (Campello et al., 2013; Ikeda et al., 2010; Liao et al., 2024; Ukita et al., 2022).

### 3.2 The UPS and circadian rhythms

The UPS is responsible for maintaining protein homeostasis by tagging proteins with ubiquitin and directing them to the 26S proteasome for degradation (Ukita et al., 2022). As the primary pathway for controlled protein turnover in eukaryotic cells, the UPS is crucial for maintaining the timing of circadian rhythms by regulating the stability of core clock proteins. In *Drosophila* and mammalian systems alike, clock proteins such as PER, tim, and CRY are rhythmically ubiquitinated and degraded, a process that defines the length of the circadian period by delaying or advancing the feedback inhibition phase (Vriend and Reiter, 2015; Ukita et al., 2022; Figure 2B). Without this tightly timed degradation, repression within the TTFL would resolve too quickly, leading to shortened circadian cycles or arrhythmicity. Thus, the UPS can serve as a molecular brake, slowing the negative feedback loop to maintain ∼24-h oscillations. Understanding how the UPS interfaces with circadian pathways reveals insights into the temporal organization of cellular processes and offers potential therapeutic targets for sleep and circadian rhythm disorders.

### 3.3 Types of ubiquitination and their involvement in circadian regulation

The specific architecture and type of ubiquitin linkage play a key role in determining the fate of the modified proteins (Yau and Rape, 2016; Kwon and Ciechanover, 2017). Ubiquitination takes on many diverse configurations and structural arrangements, and it is essential to decode such ubiquitin architectures to understand their functions.

#### 3.3.1 Monoubiquitination

Monoubiquitination, the attachment of a single ubiquitin moiety to a protein, typically regulates protein function, localization, and activity rather than signaling degradation (Lange et al., 2022; Liao et al., 2024). Core histones are key targets, with H2A monoubiquitination essential for neurodevelopment and linked to related disorders (Srivastava et al., 2017; Scheuermann et al., 2012). Similarly, H2B monoubiquitination alters chromatin structure, increasing accessibility for transcription and DNA repair, thereby promoting gene activation (Minsky et al., 2008; Fierz et al., 2011; Espinosa, 2008).

In circadian regulation, histone monoubiquitination has emerged as a dynamic and reversible mechanism that plays a key role in the rhythmic control of gene expression. Specifically, oscillations in H2B monoubiquitination have been associated with the transcriptional cycling of circadian genes, such as PER1 and PER2, indicating that chromatin remodeling through histone ubiquitination is closely aligned with the molecular clock (Tamayo et al., 2015). These modifications may act downstream of core circadian regulators or be orchestrated by ubiquitin ligases and deubiquitinating enzymes that themselves may be under circadian control.

#### 3.3.2 Polyubiquitination

Polyubiquitination is central to circadian regulation by controlling how quickly clock proteins accumulate or are degraded, thus influencing the pace and progression of the circadian cycle. K48-linked polyubiquitination, a signal for proteasomal degradation, ensures timely turnover of core clock components. For example, in *Drosophila*, the E3 ligase Cul3 promotes K48-linked ubiquitination of tim at the day–night transition, facilitating its degradation and phase progression (Szabó et al., 2018). In mammals, HRD1 mediates K48-linked degradation of BMAL1, regulating transcriptional activity of downstream circadian genes (Guo et al., 2020). These rhythms in protein turnover ensure the timely clearance of clock proteins, stabilizing period length and phase transitions.

Other polyubiquitin linkages, like K29 and K11, may modulate clock function without directly inducing degradation or indirectly via influencing pathways involved. For example, K63-linked chains affect protein interactions and chromatin recruitment (Chen et al., 2018a,Wang et al., 2017), while K11 chains may fine-tune timing and subcellular localization of clock protein degradation (Hirano et al., 2013). Together, these modifications form a dynamic ubiquitin code that regulates circadian timing and sleep-related processes by controlling the tempo of the feedback loop.

## 4 E3 ligases and deubiquitinating enzymes in circadian regulation

### 4.1 E3 ligases and their role in circadian regulation

#### 4.1.1 E3 ligases

E3 ubiquitin ligases, though structurally built on a limited set of catalytic domains, achieve functional diversity through specialized substrate recognition and regulatory elements. This modularity enables them to selectively regulate a wide array of proteins in response to cellular signals (Zheng and Shabek, 2017). With over 600 E3 ligases in humans–compared to only 2 E1s and 38 E2s–they represent the most specific step in the ubiquitin-proteasome system, playing essential roles in processes such as protein degradation, intracellular signaling, and transcriptional regulation (Zhao and Sun, 2013; Leestemaker and Ovaa, 2017; Bachiller et al., 2020). Recent evidence highlights their involvement in circadian regulation, where they modulate the stability, localization, and activity of core clock proteins (Table 1; Abdalla et al., 2022; Yang et al., 2012; Yang et al., 2014). The next several sections summarize the current status of knowledge in the field regarding E3 ligases and their roles in circadian regulation. The ligases are listed alphabetically by gene name.

**TABLE 1:** Molecular regulation of circadian clock components by E3 ubiquitin ligase enzymes or complex components.

Clock-regulated protein	Mammalian E3 ligase enzyme or complex component	Role in clock protein regulation and circadian rhythmicity	*Drosophila* ortholog	References
Clk, per, tim	TRIP12	Promotes degradation of clock proteins; knockdown increases Clk, per, tim, lengthening circadian period	ctrip	Lamaze et al., 2011; Brunet et al., 2020; Ukita et al., 2022
tim, cry	FBXL15	Mediates light-induced degradation of tim and cry for circadian resetting	jetlag	Koh et al., 2006; Peschel et al., 2006, 2009; Ozturk et al., 2011
Cry	BRWD3	CRL4 substrate receptor targeting cry for light-induced degradation; cooperates with jet and tim	BRWD3 (Ramshackle)	Jackson and Xiong, 2009; Ozturk et al., 2013; Abdalla et al., 2022
PER	–	Overexpression sustains per oscillations; may act by inhibiting cry signaling under constant light	morgue	Murad et al., 2007; Zhou et al., 2013
RDL (GABA_A receptor)	FBXL4	Promotes circadian degradation of RDL to enhance lLNv excitability and wakefulness	Fbxl4	Li et al., 2017; Parisky et al., 2008
PER, tim	β-TrCP1/β-TrCP2	Recognize and ubiquitinate Ck1-phosphorylated per proteins, promoting proteasomal degradation. Inhibition of β-TrCP–PER interaction lengthens circadian period and dampens rhythms. Knockout leads to behavioral arrhythmia under DD.	Slmb	Grima et al., 2002; Chiu et al., 2008; Eide et al., 2005
tim	CUL3	Targets hyperphosphorylated tim at day/night transitions; role in phase-shifting	CUL3	Guo et al., 2014; Szabó et al., 2018
BMAL1	UBE3A	Targets BMAL1 for degradation, reducing BMAL1-mediated transcription and rhythmicity	UBE3A	Wu et al., 2008; Gossan et al., 2014
BMAL1	HRD1	Interacts with BMAL1 and promotes its K48-linked ubiquitination without affecting mRNA; suppresses downstream PER1, DBP transcription	sip3	Guo et al., 2020; Brown, 2016; Mendoza-Viveros et al., 2017
CRY1/2, REV-ERBα	FBXL3	Degrades CRY1/2; Afh and Ovtm mutations impair this, lengthening period; also promotes REV-ERBα turnover	–	Siepka et al., 2007; Godinho et al., 2007; Shi et al., 2013
CRY1/2	FBXL21	Stabilizes CRY1/2 in the nucleus (antagonizing FBXL3), promotes degradation in cytoplasm; rhythmic SCN expression	–	Yoo et al., 2013; Dardente et al., 2008; Hirano et al., 2013
REV-ERBα	ARF-BP1, PAM	Mediate REV-ERBα degradation; knockdown leads to sustained repression of BMAL1 and disrupted clock gene oscillations	HUWE1 (ARF-BP1), highwire (PAM)	Yin et al., 2010; Stojkovic et al., 2014; Shi et al., 2013
mTOR, CRY2, REV-ERBα	FBXW7	Targets mTOR and CRY2/REV-ERBα for degradation, enabling circadian control of translation and enhancing amplitude	–	Okazaki et al., 2014; Zhao et al., 2016
DBP	TRAF7	Polyubiquitinates DBP, promoting degradation and regulating period length	–	Masuda et al., 2024
BMAL1	TRAF2	Binds BMAL1 and promotes its degradation; dampens E-box transcription and PER1 oscillation	–	Chen et al., 2018a; Rual et al., 2005
CRY1, Histones	CUL4A-DDB1-CDT2	Targets CRY1 for degradation; also mediates H2B monoubiquitination at E-boxes for chromatin remodelling	CUL4	Tong et al., 2015, 2017; Tamayo et al., 2015
BMAL1, PER2	UBE2O	Binds BMAL1 and promotes degradation; its silencing increases PER2 rhythm amplitude	–	Chen et al., 2018b; Ullah et al., 2019
BMAL1	STUB1 (CHIP)	Binds and degrades BMAL1 via K48 ubiquitination; translocates to nucleus under stress to modulate BMAL1 and senescence	STUB1	Ullah et al., 2020; VanPelt and Page, 2017
PER2	MDM2	Ubiquitinates PER2 to regulate its degradation; PER2 stabilizes p53 by blocking MDM2	–	Liu et al., 2018; Gotoh et al., 2015; Fagiani et al., 2022
REV-ERBα, REV-ERBβ	SIAH2	Promotes REV-ERB degradation; knockout alters circadian period and shows female-specific liver rhythm shifts	–	DeBruyne et al., 2015; Mekbib et al., 2022

#### 4.1.2 ARF-BP1, PAM and FBXL3

ARF-BP1, also known as HUWE1 in *Drosophila*, and PAM, also known as highwire in *Drosophila*, are critical E3 ubiquitin ligases that regulate the stability of REV-ERBα, a component of the circadian clock mentioned above that is responsible for repressing genes like BMAL1 (Yin et al., 2010). These E3 ligases associate with REV-ERBα and mediate its ubiquitination, targeting it for proteasomal degradation following circadian cues such as lithium treatment or serum shock, both of which synchronize circadian oscillations in mammalian cells (Stojkovic et al., 2014; Yin et al., 2010). Knockdown of ARF-BP1 or PAM in mouse hepatoma cells stabilizes REV-ERBα protein preventing its timely degradation, which results in sustained repression of BMAL1 and disruption of oscillations in multiple other clock genes (Yin et al., 2010).

FBXL3, beyond targeting CRY proteins, also indirectly influences REV-ERBα-mediated repression of *BMAL1* and *CRY1*, as elevated REV-ERBα levels in *FBXL3*-mutant mice enhance transcriptional repression (Shi et al., 2013; Stojkovic et al., 2014). Deletion of *REV-ERBA* in the *FBXL3*-mutant mice rescues the mutant circadian phenotype, highlighting FBXL3’s broader role in coordinating core clock transcription factors (Shi et al., 2013). Together, these findings demonstrate that ARF-BP1, PAM and FBXL3 are critical E3 ligases that regulate REV-ERBα degradation to maintain the stability and timing of circadian clock gene expression (Shi et al., 2013; Stojkovic et al., 2014; Yoo et al., 2013).

#### 4.1.3 BRWD3

BRWD3 (Bromodomain and WD Repeat Domain Containing 3), also known as ramshackle, acts as a substrate receptor for the Cul4-RING E3 ubiquitin ligase complexes and mediates light-induced degradation of cry (Jackson and Xiong, 2009; Ozturk et al., 2013). Identified through a yeast two-hybrid assay, BRWD3 knockdown strongly attenuated light-dependent cry degradation in S2 cells (Ozturk et al., 2013). *In vivo* assays confirmed that BRWD3 binds cry in a light-dependent manner and, as a part of the BRWD3-Cullin4-RING Finger E3 Ligase (CRL4) complex, catalyzes cry ubiquitination and subsequent proteasomal degradation (Ozturk et al., 2013). BRWD3 also co-precipitated with other CRL4 E3 ligase components such as Damage-specific DNA binding protein 1 (DDB1), Cul4, and Regulator of cullins 1a (Roc1). These findings establish BRDW3 as a light-dependent cry receptor (Ozturk et al., 2013). Together, BRWD3 and jet appear to act cooperatively during photic resetting, where light exposure promotes the formation of a complex including tim, cry, jet, and BRWD3, enabling coordinated degradation of both tim and cry (Ozturk et al., 2013; Abdalla et al., 2022).

#### 4.1.4 ctrip

circadian trip (ctrip) is a HECT-type E3 ubiquitin ligase in *Drosophila*, with sequence homology to mammalian TRIP12 (Brunet et al., 2020). Although *ctrip* mRNA shows no clear circadian oscillations, its expression is highly enriched in pigment-dispersing factor (Pdf)-positive lateral ventral neurons (LN_*v*_s), key pacemaker neurons in the fly brain (Lamaze et al., 2011; Ukita et al., 2022). Functional studies demonstrate that ctrip is a regulator of molecular behavioral rhythms. Deletion of the N-terminal exons of *ctrip* disrupts larval clock function, resulting in elevated levels of per, tim, and Clock in the small LN_*v*_s and lengthened period of protein oscillations (Lamaze et al., 2011). Similarly, RNAi-mediated knockdown of *ctrip* in *tim*-expressing adult clock cells slows locomotor activity rhythms and elevates Clk protein abundance, indicating ctrip’s role in promoting Clk degradation (Lamaze et al., 2011). Moreover, downregulation of *ctrip* leads to the persistence of phosphorylated per and tim during the subjective day, suggesting impaired degradation of these repressors as well. In *per*-null flies, *ctrip* knockdown still increases Clk protein levels but has no effect on tim, implying that ctrip may regulate Clk and per independently from tim (Lamaze et al., 2011).

#### 4.1.5 Cullin-3 (Cul3)

In *Drosophila*, Cul3, a member of the Cullin E3 ligase family, plays a significant role in circadian regulation (Guo et al., 2014). Targeted knockdown of *Cul3* in clock neurons disrupts morning anticipation and induces arrhythmic behavior under constant darkness (DD), while pan-neuronal knockdown causes complete lethality, highlighting its broader essential importance (Grima et al., 2012). Cul3 associates with hyperphosphorylated tim, promoting its K48-linked ubiquitination and degradation, particularly at the day–night transition (Guo et al., 2014; Szabó et al., 2018). This timing is crucial as premature degradation of tim in late night results in phase advances, whereas early night degradation leads to phase delays (Guo et al., 2014). Cul3 also mediates light-induced phase resetting in cry-deficient flies, suggesting it participates in photic signaling pathways independent of cry (Ogueta et al., 2020).

#### 4.1.6 CUL4A-DDB1-CDT2

The CUL4A-DDB1-CDT2 E3 ubiquitin ligase complex is an important regulator of the mammalian circadian clock, acting through both protein turnover and chromatin modifications. This complex promotes the ubiquitin-mediated degradation of CRY1, specifically targeting lysine 585, thereby limiting CRY1 accumulation and constraining the amplitude of circadian oscillations (Tong et al., 2015; Tong et al., 2017). In parallel, CLOCK-BMAL1 recruits CUL4A-DDB1 to circadian target genes such as PER1, PER2, and CRY1, where it catalyzes rhythmic histone H2B monoubiquitination at E-box sites (Tamayo et al., 2015). This chromatin mark facilitates the recruitment of the repressive PER complex, establishing a feedback mechanism that links the positive and negative limbs of the clock (Tamayo et al., 2015). Loss of DDB1-CUL4 or disruption of H2B monoubiquitination impairs this recruitment and weakens transcriptional feedback, underscoring the dual role of this E3 ligase complex in regulating clock protein stability and feedback timing (Tamayo et al., 2015; Tong et al., 2017; Tong et al., 2015).

#### 4.1.7 Fbxl4

Fbxl4 is a Clock-regulated E3 ubiquitin ligase that links molecular circadian oscillators to neuronal excitability and sleep timing in *Drosophila* (Li et al., 2017). *Fbxl4* transcript levels cycle rhythmically in the large ventral lateral neurons (lLNvs) and are driven directly by Clock-cycle binding to E-box elements within the fbxl4 promoter (Li et al., 2017). Fbxl4 promotes the rhythmic ubiquitination and degradation of GABA_A_ receptors in lLNvs, thereby reducing GABA sensitivity and enhancing neuronal excitability during the wake phase (Li et al., 2017). Loss of fbxl4 disrupts circadian control of sleep by enhancing GABAergic inhibition in arousal-promoting lLNvs. Fbxl4 mutant flies exhibit increased daytime and nighttime sleep and shortened sleep onset latency, similar to flies overexpressing the GABA receptor RDL (Parisky et al., 2008; Agosto et al., 2008). These findings indicate that Fbxl4 promotes wakefulness by rhythmically degrading RDL in a clock dependent manner, linking the circadian clock to sleep timing.

#### 4.1.8 FBXL3 and FBXL21

FBXL3 and FBXL21 are closely related F-box proteins that regulate circadian rhythms by modulating the stability and degradation of CRY1 and CRY2 proteins in mammals (Abdalla et al., 2022; Busino et al., 2007). Forward genetic screens in mice identified two mutations in FBXL3–C358S (Afterhours, Afh) and I364T (Overtime, Ovtm)–that significantly lengthen circadian period (Siepka et al., 2007). These mutations impair FBXL3’s interaction with CRY proteins and reduce its catalytic efficiency, leading to CRY1/2 stabilization and strong suppression of E-box-driven transcription of PER genes (Godinho et al., 2007; Siepka et al., 2007). Interestingly, the long-period phenotype in FBXL3 mutants is rescued by REV-ERBα deletion, indicating that FBXL3 also promotes circadian period determination and clock robustness by influencing REV-ERBα stability and transcriptional activity (Shi et al., 2013).

Further genetic studies revealed the *Past-time (Psttm)* mutation in FBXL21 shortens circadian period and counteracts the long-period phenotype of *FBXL3 (Ovtm)*, illustrating their functional antagonism (Yoo et al., 2013). It was shown in mice that FBXL21 exhibits strong, SCN-restricted expression with pronounced diurnal and circadian rhythmicity, rising rapidly at the start of the day and declining at night–a pattern reminiscent of other CLOCK:BMAL1-driven genes such as *PER1* and *DBP (D-site binding protein)* (Dardente et al., 2008). Although FBXL21 also binds to CRY proteins and promotes their ubiquitination, FBXL21 serves a dual role: in the nucleus, it antagonizes FBXL3 by binding and stabilizing CRY1/2, protecting them from degradation; in the cytoplasm, where FBXL3 is largely absent, FBXL21 promotes slow degradation of CRY proteins (Yoo et al., 2013). Collectively, these findings establish FBXL21 as a rhythmically expressed, clock-controlled gene that modulates circadian period of the clock by regulating CRY protein stability in opposition to FBXL3, with distinct roles in nuclear and cytoplasmic compartments (Yoo et al., 2013; Dardente et al., 2008; Hirano et al., 2013).

#### 4.1.9 FBXW7

FBXW7 is an E3 ubiquitin ligase that plays a role in regulating circadian rhythms by targeting core clock proteins and modulators for degradation. The expression of FBXW7 itself is circadian and regulated by the transcription factor DBP, which rhythmically activates FBXW7 transcription in renal tumor models (Okazaki et al., 2014). FBXW7 contributes to circadian rhythmicity by targeting mTOR, a key regulator of circadian translation, for ubiquitin-mediated degradation, resulting in opposing oscillations between FBXW7 and mTOR protein levels and enabling rhythmic control of translational output (Okazaki et al., 2014; Cao, 2018). FBXW7 also directly modulates clock repressors CRY2 and REV-ERBα for degradation via a CDK1-dependent phosphorylation site (Thr275), relieving repression of the positive arm of the clock and enhancing circadian amplitude (Zhao et al., 2016). Hepatic deletion of *FBXW7* disrupts rhythmic expression of core clock genes and alters systemic metabolic outputs, including whole-body lipid and glucose homeostasis (Zhao et al., 2016). Together, these findings establish FBXW7’s roles as a post-translational regulator of the circadian clock, modulating negative feedback repressors and linking metabolic signals to molecular oscillators.

#### 4.1.10 HRD1

The E3 ubiquitin ligase HRD1, encoded by the Synoviolin 1 gene, has been identified as a post-translational regulator of the core circadian transcription factor BMAL1 in mammals. Co-immunoprecipitation and immunofluorescence assays demonstrated that HRD1 physically interacts with BMAL1 and enhances its K48-linked polyubiquitination, leading to proteasome-mediated degradation without altering BMAL1 mRNA levels (Guo et al., 2020). BMAL1 forms heterodimers with CLOCK to drive the transcription of core clock-controlled genes such as PER’S, CRY’S and DBP (Brown, 2016; Mendoza-Viveros et al., 2017), and consistently, *HRD1* overexpression significantly reduces PER1 and DBP mRNA levels, suggesting downstream transcriptional suppression via BMAL1 destabilization (Guo et al., 2020). Further supporting this, luciferase assays revealed that while HRD1 does not affect BMAL1 promoter activity, it significantly represses PER1 promoter activity in the presence of BMAL1 and CLOCK, suggesting that HRD1 regulates the expression of PER1 and DBP indirectly by reducing BMAL1 protein levels (Guo et al., 2020). A role for the *Drosophila* ortholog of HDR1, called septin interacting protein 3 (sip3), in circadian regulation has not been identified.

#### 4.1.11 jetlag

jetlag, mentioned previously, is an F-box protein with leucine-rich repeats that functions as a substrate adaptor within SCF-type E3 ubiquitin ligase complexes and plays a critical role in light-dependent resetting of the *Drosophila* circadian clock (Koh et al., 2006; Peschel et al., 2006). Initially identified through mutant screens for flies exhibiting persistent rhythmic behavior under constant light, jet was found to mediate the photic degradation of the core clock protein timeless, thereby facilitating circadian entrainment (Koh et al., 2006; Peschel et al., 2009). Loss-of-function jet mutants show impaired phase shifts in response to light pulses, slower re-entrainment to shifted light-dark cycles, and stabilized tim protein levels under light conditions (Koh et al., 2006). These phenotypes were rescued by reintroducing wild-type *jet* in clock neurons. In cultured cells, co-expression of *jet* and *cry* is sufficient to recapitulate light-induced degradation of tim, suggesting that jet transduces cry-dependent light input to tim (Koh et al., 2006).

Beyond tim, jet has also been shown to mediate light-induced degradation of cry itself, indicating a broader role in light-responsive circadian protein turnover (Peschel et al., 2009). Yeast two-hybrid and *in vivo* studies demonstrate that jet binds to cry in a light-induced manner, and that jet deficiency leads to cry accumulation in fly heads and cultured cells (Peschel et al., 2009). The relative abundance of tim influences cry stability, as tim competitively inhibits cry degradation, indicating that jet targets these proteins sequentially depending on their relative affinities (Peschel et al., 2009). Structural studies have further revealed that light exposure induces a conformational change in cry that enhances its interaction with jet, enabling ubiquitin-mediated degradation (Peschel et al., 2009; Ozturk et al., 2011). Collectively, these findings establish jet as a central component of the UPS in circadian phototransduction, regulating the timely degradation of both tim and cry to ensure precise clock resetting in response to environmental light cues.

#### 4.1.12 MDM2

The E3 ligase MDM2 ubiquitinates PER2 at conserved lysine residues in mammalian cells, promoting its proteasomal degradation and regulating circadian period length (Liu et al., 2018). MDM2 is a well-known oncogene, frequently overexpressed in cancers where it promotes degradation of the tumor suppressor p53 (Lee and Gu, 2010; Cheok et al., 2011). PER2 forms complexes with both MDM2 and p53 but can also bind MDM2 independently (Liu et al., 2018). Importantly, the circadian interplay between PER2 and p53 forms a regulatory feedback loop in which p53 suppresses PER2 expression, while PER2 stabilizes p53 by inhibiting MDM2-dependent ubiquitination, linking circadian rhythm to cellular stress responses and tumor suppression (Gotoh et al., 2015; Fagiani et al., 2022).

#### 4.1.13 morgue

morgue is a unique ubiquitin pathway protein in *Drosophila* notable for its dual-domain structure containing an F-box and a non-catalytic E2-conjugase domain (Hays et al., 2002; Wing et al., 2002). It physically associates with SkpA, a core component of SCF-type E3 ligases, and binds K48-linked polyubiquitin chains, implicating it in proteasomal degradation (Zhou et al., 2013). In the circadian clock, morgue has emerged as a regulator of light input and behavioral rhythmicity under constant light. Overexpression of morgue in the tim-expressing neurons, but not in Pdf-expressing neurons, confers resistance to constant light-induced arrhythmicity and sustains robust per oscillations, specifically in DN1 neurons (Murad et al., 2007). This suggests that morgue supports rhythmicity in a neuron-specific manner, independently of the canonical LNv pacemakers. Moreover, behavioral phase shifts to light are blunted in morgue-overexpressing flies, reminiscent of cry hypomorphs, pointing to a possible inhibitory role for morgue in the cry signaling pathway (Murad et al., 2007; Busza et al., 2004).

#### 4.1.14 SIAH2

SIAH2 (Seven in Absentia 2) is a RING-type E3 ubiquitin ligase that promotes the ubiquitination and proteasomal degradation of REV-ERBα and REB-ERBβ, two core repressors in the circadian clock (DeBruyne et al., 2015). In cultured cells, SIAH2 loss stabilizes REV-ERBs, disrupting rhythmicity, altering downstream gene expression, and lengthening the circadian period, highlighting the role of SIAH2 in clock timing via UPS-mediated turnover (DeBruyne et al., 2015). In vivo, *SIAH2* deletion modestly affects expression of other clock genes such as BMAL1 and PER2 but does not significantly alter REV-ERBα protein rhythms, likely due to compensatory E3 ligases (Mekbib et al., 2022). Interestingly, *SIAH2* deficiency in female mice leads to a phase-advanced circadian liver transcriptome and altered lipid rhythms, revealing an intriguing sex-specific role for UPS regulation in circadian rhythms, whose cause remains unknown (Mekbib et al., 2022).

#### 4.1.15 Supernumerary limbs (slmb) and β-TrCP1 and β-TrCP2

The *Drosophila* Cullin-RING E3 ligase component slmb, encoded by *slmb*, and its mammalian orthologs β-TrCP1 and β-TrCP2 (F-box proteins within the SCF complex) are critical regulators of circadian timing through their control of per and tim protein stability (Grima et al., 2002; Shirogane et al., 2005). In flies, slmb targets phosphorylated per for ubiquitin-mediated degradation, a process initiated by dbt-dependent phosphorylation at Ser47 (Chiu et al., 2008; Grima et al., 2002). *Slmb* mutants fail to anticipate light-to-dark transitions under light/dark (LD) conditions and display completely arrhythmic behavior under constant darkness (DD), indicating disrupted circadian control (Grima et al., 2002). Remarkably, rhythmicity was rescued by restoring *slmb* in Pdf-positive neurons, highlighting its essential role in the clock network (Grima et al., 2002). Interestingly, slmb expression is not rhythmic, indicating how non-rhythmic E3 ligases can still exert strong influence over rhythmic biological processes. For instance, although slmb levels remain constant, its activity becomes critical at specific phases of the cycle such as late night when degradation of per and tim relieves transcriptional repression and resets the clock (Grima et al., 2002; Ko et al., 2002). Slmb’s constant presence may allow it to act flexibly at multiple points in the cycle, illustrating how stable components of the UPS can contribute to temporal regulation in a circadian context.

In mammals, β-TrCP1 and β-TrCP2 similarly recognize, and target phosphorylated PER proteins, particularly following casein kinase 1 (CK1) phosphorylation, for degradation (Eide et al., 2005). Both β-TrCP1 and β-TrCP2 bind PER1/2, and expression of a mutant β-TrCP lacking the F-box domain prevents PER2 degradation (Eide et al., 2005; Shirogane et al., 2005). Disruption of β-TrCP–PER interactions in cells lengthens period or dampens rhythms, while inducible knockout of β-TrCP2 in mice leads to disrupted behavioral rhythms and variable period lengths under DD (Reischl et al., 2007; D’Alessandro et al., 2017). β-TrCP1/2 also regulates the degradation of other circadian proteins such as DEC1, further emphasizing their broad role in maintaining circadian period and amplitude (Kim et al., 2014).

#### 4.1.16 STUB1

STIP1 homology U-box-containing protein 1 STUB1, also known as C terminus of HSP70-interacting protein (CHIP), is a multifunctional protein with both chaperone activity and U-box-dependent E3 ubiquitin ligase activity (Ballinger et al., 1999; Rosser et al., 2007). STUB1 plays a central role in protein quality control by linking molecular chaperones to the UPS, playing a critical role in proteostasis (VanPelt and Page, 2017). It targets a range of substrates, including misfolded or oxidized proteins, tau, polyglutamine-expanded proteins, and key signaling factors (Lee et al., 2013; Jana et al., 2005; Seo et al., 2016; Xin et al., 2005; Hatakeyama et al., 2004; Murata et al., 2001). In circadian regulation, STUB1 was identified as a selective BMAL1-binding protein in a mass spectrometry screening, with no observed interaction with CLOCK, indicating a specific role in targeting BMAL1 (Ullah et al., 2020). Overexpression of wild-type *STUB1*, but not of a catalytically inactive mutant, leads to reduced BMAL1 protein levels, confirming its role in regulating BMAL1 stability through K48-linked polyubiquitination and proteasomal degradation (Ullah et al., 2020). Under oxidative stress, STUB1 translocates to the nucleus where it enhances BMAL1 degradation and modulates circadian-linked cellular senescence (Ullah et al., 2020). These findings underscore STUB1 as a post-translational regulator of the clock via linking the UPS to circadian timing and stress adaptation.

#### 4.1.17 TRAF7

Tumor Necrosis Factor Receptor-Associated Factor 7 (TRAF7), a RING-type E3 ubiquitin ligase, has recently been identified as a key regulator of circadian timing through its modulation of DBP, a clock-controlled transcription factor that drives rhythmic gene expression via D-box elements (Masuda et al., 2024). DBP exhibits strong circadian oscillation at the protein level, and this rhythmicity is shown to be regulated post-translationally through K48-linked polyubiquitination by TRAF7, in cooperation with E2 enzymes UBE2G1 and UBE2T. In cells, Masuda et al. (2024) revealed overexpression of *TRAF7* promotes DBP degradation, while *TRAF7* knockdown up-regulated DBP and disrupts its time-of-day–dependent oscillation. TRAF7 also shortens the circadian period, underscoring its broader role in shaping clock dynamics (Masuda et al., 2024). These findings position TRAF7 as an important link between the UPS and circadian regulation through its targeted destabilization of DBP.

In addition to TRAF7, the E3 ligase TRAF2 has also been implicated in clock protein regulation. Initially identified as a CRY1-interacting protein in a high-throughput yeast two-hybrid screen (Rual et al., 2005), TRAF2 was later shown to bind directly to BMAL1, reducing its abundance without affecting CRY1 levels (Chen et al., 2018a). This interaction is mediated though TRAF2’s zinc finger domain rather than the canonical TRAF domain, and deletion of its RING domain stabilized BMAL1, confirming the importance of its ubiquitin ligase activity (Chen et al., 2018a). *TRAF2* overexpression enhances BMAL1 ubiquitination and proteasomal degradation, thereby attenuating E-box-mediated transcription and dampening PER1 oscillations (Chen et al., 2018a). Together, TRAF7 and TRAF2 highlight how multiple TRAF family E3 ligases influence circadian output by targeting distinct transcriptional regulators.

#### 4.1.18 UBE3A

UBE3A, an E3 ubiquitin ligase previously implicated in Angelman syndrome and HPV-related tumorigenesis, has recently emerged as a conserved regulator of the circadian clock through its post-translational control of BMAL1 stability (Gossan et al., 2014; Mabb et al., 2011). Experimental activation of *UBE3A*–achieved via expression of the HPV E6/E7 oncogenes–in murine fibroblasts significantly disrupted circadian oscillations, reduced BMAL1 protein levels, and increased BMAL1 ubiquitination (Gossan et al., 2014). These effects were dependent on UBE3A’s ligase activity and were observed both with and without oncogene expression, indicating an endogenous role for UBE3A in clock regulation.

In *Drosophila*, both overexpression and knockdown of *Ube3a* in central clock neurons resulted in pronounced alterations in circadian locomotor rhythms, indicating that tight regulation of *Ube3a* levels is essential for proper pacemaker function across species (Gossan et al., 2014; Wu et al., 2008). Furthermore, flies harboring a global null mutation of *Ube3a* exhibited significant circadian deficits, supporting the notion that Ube3a is an integral component of the *Drosophila* circadian clock (Wu et al., 2008). These findings, together with the widespread expression of UBE3A in mammalian tissues including the SCN, suggest a conserved role for UBE3A in circadian timekeeping across species (Tian et al., 2011; Wu et al., 2008; Gossan et al., 2014).

#### 4.1.19 UBE2O

UBE2O, a unique ubiquitin-conjugating enzyme with hybrid E2/E3 ligase activity (Ullah et al., 2019), has emerged as a regulator of the circadian clock. In HEK293T cells, *UBE2O* overexpression leads to a dose-dependent reduction of endogenous BMAL1, while its knockdown increases BMAL1 protein levels (Chen et al., 2018b). UBE2O physically associates with BMAL1 in mouse Neuro2a cells and whole brain tissue, but does not associate with CLOCK, demonstrating its specificity for BMAL1 (Chen et al., 2018b). Functionally, *UBE2O*overexpression attenuates BMAL1-driven transcription and dampens downstream circadian outputs, while its silencing increases *PER2* rhythm amplitude, confirming its role in maintaining clock dynamics (Chen et al., 2018b). Beyond circadian regulation, UBE2O dysfunction is implicated in diseases such as cancer, diabetes, and obesity, linking its disruption to broader physiological consequences (Maffeo and Cilloni, 2024).

### 4.2 Deubiquitinating enzymes and their role in circadian regulation

#### 4.2.1 Deubiquitinating enzymes

Deubiquitinases (DUBs) are enzymes that remove ubiquitin moieties from target proteins, reversing the action of E3 ubiquitin ligases and thereby shaping ubiquitin signaling with high accuracy (Srikanta and Cermakian, 2021; Stojkovic et al., 2014). In circadian biology, DUBs serve as key modulators of clock protein stability, ensuring the robustness and proper timing of transcriptional-translational feedback loops that drive 24-h rhythms (Harris-Gauthier et al., 2022). Recent work has highlighted their emerging importance in both central and peripheral clocks, where they fine-tune the amplitude, period, and phase of circadian gene expression (Harris-Gauthier et al., 2022; Table 2). We summarize the current status of knowledge in the field regarding DUB involvement in circadian regulation in the next few sections, with DUBs listed alphabetically by gene name.

**TABLE 2:** Molecular regulation of circadian clock components by deubiquitinating enzymes.

Clock regulated protein affected	Mammalian DUB	Role in modulating clock protein stability and circadian rhythmicity	*Drosophila* ortholog	References
CRY1, CRY2, BMAL1, PER1	USP2	USP2 is rhythmically expressed and modulates CRY1, BMAL1, and PER1 stability; its deletion disrupts central and peripheral rhythms and impairs SCN synchrony.	USP2	Harris-Gauthier et al., 2022; Scoma et al., 2011; Tong et al., 2012; Yang et al., 2014; Srikanta et al., 2025
CRY1, CRY2	USP7	Deubiquitinates and stabilizes CRY1/2. Knockdown alters period length; mediates response to genotoxic stress. Negatively regulated by MAGEL2.	USP7	Papp et al., 2015; Hirano et al., 2016; Carias et al., 2020
CLOCK, BMAL1, PER2, CRY1, CRY2	MAGEL2 (regulatory partner, not a DUB itself)	Regulates CLOCK/BMAL1/PER2 via interactions with USP7/USP8 and E3 ligases. Modulates CRY1 stability and circadian output.	–	Devos et al., 2011; Hirano et al., 2016; Carias et al., 2020; Kozlov et al., 2007
BMAL1	USP1	Stabilizes BMAL1 protein post-transcriptionally. Knockdown reduces BMAL1 and target genes. Dominant in heart tissue.	USP1	Hu et al., 2024
BMAL1	USP13	Stabilizes BMAL1 by preventing its degradation. Loss impairs rhythmic BMAL1 oscillation.	–	Gu et al., 2025
PER1, PER2	USP14	Stabilizes PER proteins. Knockdown shortens period. Downregulation corrects circadian defects in Parkinson’s disease fly model.	Usp14	D’Alessandro et al., 2017; Favaro et al., 2024
BMAL1	USP9X	Stabilizes BMAL1 via deubiquitination. Knockdown alters target gene expression. Tissue-specific effects on rhythm amplitude.	Faf	Zhang et al., 2018
Clock	USP8	Deubiquitinates clock to suppress clock/cycle transcriptional activity. Knockdown disrupts behavioral rhythms.	USP8	Luo et al., 2012; Kula-Eversole et al., 2010; McDonald and Rosbash, 2001
tim, per, Clock, Pdp1ε	USP22	Part of SAGA complex. Knockdown increases H2B ubiquitination, lowers clock gene expression, and lengthens period.	non-stop (not)	Mahesh et al., 2020; Bu et al., 2020; Tamayo et al., 2015

#### 4.2.2 MAGEL2

MAGEL2 plays an important role in modulating clock protein ubiquitination by acting in concrete with both E3 ligases and deubiquitinases. Among its known binding partners are E3 ligases TRIM27 and RNF41, and the deubiquitinases USP7 and USP8, which it recruits to regulate the ubiquitination and stability of diverse targets (Tacer and Potts, 2017; Hao et al., 2013; Wijesuriya et al., 2017). MAGEL2 was shown to modulate the stability and activity of key circadian regulators including CLOCK, BMAL1, and PER2 (Devos et al., 2011). In addition, MAGEL2 modulates the ubiquitination and stability of CRY1 and CRY2 through interactions with USP7 and FBXL proteins (Hirano et al., 2016; Ansar et al., 2019). MAGEL2, in cooperation with E3 ligases and deubiquitinases, fine-tunes CRY1 protein levels through a ubiquitin-mediated degradation pathway, contributing to the temporal control of CRY1 stability and ensuring proper progression of the negative feedback loop that sustains circadian rhythms (Carias et al., 2020).

The biological importance of MAGEL2-mediated regulation of clock proteins is underscored by the circadian phenotypes observed in *MAGEL2* knock-out mice. These animals exhibit fragmented activity and mistimed daytime behavior under light/dark cycles (Kozlov et al., 2007), supporting a role for MAGEL2 in maintaining circadian output. *MAGEL2* mRNA is rhythmically expressed in the SCN, particularly in vasopressin-expressing neurons that are critical for circadian outputs, and this rhythmicity persists even in constant darkness (Kozlov et al., 2007). Moreover, *MAGEL2* expression is disrupted in mice carrying mutations in the core circadian regulator *CLOCK* (Carias et al., 2020). *MAGEL2* is the only gene among those disrupted in Prader-Willi syndrome with expression and function linked to excessive daytime sleepiness and night waking, and while it is also mutated in Schaaf-Yang syndrome, circadian features in this disorder remain poorly characterized due to the small number of diagnosed individuals (Fountain et al., 2017; Schaaf et al., 2013). Together, these findings highlight MAGEL2 and its interaction with E3 ligases and deubiquitinases in circadian rhythm control.

#### 4.2.3 USP1

A genome-wide CRISPR/Cas9 knockdown screen identified ubiquitin-specific protease 1 (*USP1*) as a novel regulator that positively modulates BMAL1 protein levels (Hu et al., 2024). Overexpression of wild-type *USP1*, but not its catalytically inactive mutant, led to increased BMAL1 protein abundance, whereas *USP1* knockdown via shRNA or CRISPR knockout significantly reduced BMAL1 levels in U2OS cells (Hu et al., 2024). Pharmacological inhibition of USP1 similarly reduced BMAL1 protein levels both *in vitro* and in mouse tissues, further confirming its role in regulating BMAL1 stability (Hu et al., 2024). USP1 modulates BMAL1 at the post-transcriptional level, as its silencing did not affect *BMAL1* mRNA levels. Furthermore, reduced levels or inhibition of USP1 resulted in decreased expression of several BMAL1 target genes, including *CRY1, CRY2, PER1, PER2*, and *DBP*, along with reduced protein levels of BMAL1, CRY1, and CRY2 (Hu et al., 2024).

Interestingly, *USP1* displays a strong tissue-specific expression. In the mouse heart, *USP1* expression is markedly higher than the other *BMAL1* deubiquitinases, *USP2* and *USP9X*, while in the hypothalamus *USP9X* was much higher than the others (Hu et al., 2024). This suggests that distinct DUBs may control BMAL1 stability in a tissue-specific manner, with *USP1* playing a dominant role in cardiac circadian regulation.

#### 4.2.4 USP2

One of the most well characterized DUBs in circadian biology is USP2. It is rhythmically expressed across multiple tissues including the suprachiasmatic nucleus (SCN), liver, and retina and its deletion disrupts clock gene expression in both central and peripheral oscillators (Harris-Gauthier et al., 2022; Scoma et al., 2011). USP2 exists in multiple isoforms, with evidence that USP2a stabilizes CRY1, while USP2b interacts with BMAL1 and PER1, modulating their localization and stability (Scoma et al., 2011; Tong et al., 2012).

Cellular overexpression of *USP2* enhances PER1-mediated repression on CLOCK/BMAL1 transcriptional activity by promoting PER nuclear retention, and consistently, USP2 knockout mice exhibit increased cytoplasmic PER1 and dampened PER2 and REV-ERBα rhythmicity (Yang et al., 2014). More recently, Srikanta and Cermakian (2021), Srikanta et al. (2025) demonstrated that *USP2* deletion in mice impairs synchrony among SCN neurons, dampens molecular rhythms, and alters behavioral circadian outputs, reinforcing USP2’s role in maintaining coherence within the central clock network (Srikanta et al., 2025).

#### 4.2.5 USP7

USP7, also known as HAUSP (Herpes virus-associated ubiquitin-specific protease), has been implicated in circadian rhythms by stabilizing CRY1 and CRY2 proteins through deubiquitination (Hirano et al., 2016; Papp et al., 2015). While reducing USP7 activity via RNA interference or pharmacological inhibition lengthened circadian period in MEFs and U2OS cells (Papp et al., 2015), other studies reported the opposite effect, with knockdown shortening and overexpression lengthening the period (Hirano et al., 2016). These discrepancies may stem from cell type differences, CRY paralog-specific roles, or redundancy with other DUBs (Maier et al., 2009; Baggs et al., 2009).

Importantly, Papp et al. (2015) demonstrated that genotoxic stress induces CRY1 phosphorylation and USP7-mediated stabilization, shifting clock phase and increasing the CRY1/CRY2 ratio, which helps coordinate circadian and DNA damage responses (Papp et al., 2015). USP7 itself is negatively regulated by MAGEL2, an E3 ligase mentioned above which is highly expressed in the SCN and associated with sleep disorders and circadian rhythm disruptions in both humans and mice (Carias et al., 2020; Kozlov et al., 2007; Lee et al., 2003), suggesting that USP7 may influence mammalian sleep–wake regulation as part of a broader transcriptional complex. Circadian roles for the fly *Usp7* have not yet been defined.

#### 4.2.6 USP13

Recent studies have revealed a link between ubiquitin-specific protease 13 (USP13) and circadian rhythm regulation through its interaction with BMAL1. USP13 was identified as a downstream effector of TDP-43, a protein implicated in several neurodegenerative disorders including amyotrophic lateral sclerosis (ALS), frontotemporal dementia (FTD), and Alzheimer’s disease (AD) (Chen and Mitchell, 2021; Josephs et al., 2014; Neumann et al., 2006). TDP-43 exhibits rhythmic expression, and its knockdown disrupts circadian behavior, alters the expression of core clock genes including BMAL1, CLOCK, CRY1, and PER2, and weakens cognition and balancing abilities in mice (Gu et al., 2025). Mechanistically, Gu et al. (2025) found *TDP-43* knockdown induces aberrant splicing and downregulation of USP13, which in turn leads to increased ubiquitination and degradation of BMAL1. Increasing the amount of *USP13* in HEK-293T cells increased BMAL1 protein in a dose-dependent manner, supporting USP13s role in BMAL1 stabilization via directly modulating its ubiquitination (Gu et al., 2025). Furthermore, USP13 itself is rhythmically expressed, and disruption of its expression perturbs the normal oscillation of BMAL1 protein levels (Gu et al., 2025). These findings position USP13 as a key post-translational regulator, linking deubiquitination of BMAL1 to broader rhythms in physiology and highlighting its potential as a molecular target in circadian dysfunction associated with neurodegenerative conditions.

#### 4.2.7 Usp8

In *Drosophila*, DUBs such as Usp8 and non-stop (USP22 in humans) have been shown to influence circadian rhythms through transcriptional regulation. Usp8, identified as rhythmically expressed in the *Drosophila* brain and more specifically clock neurons, directly deubiquitinates Clock to suppress its transcriptional activity within the Clock-cycle complex, thereby reducing expression of target genes like *per* and *tim* (McDonald and Rosbash, 2001; Kula-Eversole et al., 2010; Luo et al., 2012). Flies with *Usp8* knockdown exhibited disrupted locomotor activity rhythms and displayed either arrhythmic or lengthened periods, displaying the role of Usp8 in maintaining circadian timing (Luo et al., 2012). There is currently no reported role for mammalian USP8 in circadian regulation.

#### 4.2.8 USP9X and USP14

USP9X has been implicated in regulation of the positive arm of the molecular clock by deubiquitinating and stabilizing BMAL1 (Zhang et al., 2018). Reducing USP9X lowered the expression of BMAL1 target genes PER2 and CRY1 in mouse neuroblastoma cells, but in U2OS cells, knockdown only slightly reduced rhythm amplitude without changing the period (Zhang et al., 2018). These differences may reflect tissue-specific interactions or compensation by other DUBs, highlighting the need for further study of USP9X and other DUBs roles in circadian regulation across tissues.

USP14 has been implicated in stabilizing PER proteins, as expression of a dominant-negative *USP14* in HEK293 cells decreases levels of PER1 and PER2, and in MEFs leads to reduced PER2 half-life and a dose-dependent shortening of circadian period, indicating USP14’s potential role in regulating the timing of the negative feedback loop (D’Alessandro et al., 2017; D’Alessandro et al., 2015). Interestingly, a recent study showed *Usp14* down-regulation corrects sleep and circadian dysfunction of a *Drosophila* model of Parkinson’s disease, suggesting it may modulate neurodegeneration-related disruptions in biological time (Favaro et al., 2024).

#### 4.2.9 non-stop and Nipped-A

non-stop (not; also known as USP22 in humans), the deubiquitinase module of the Spt-Ada-Gcn5 acetyltransferase (SAGA) chromatin-modifying complex, modulates transcription through histone H2B deubiquitination (Mohan et al., 2014). *not* knockdown in clock neurons lengthened locomotor periods, reduced rhythm robustness, and decreased expression of several clock genes, including *tim*, *per*, and *Clock* (Mahesh et al., 2020; Bu et al., 2020). Furthermore, loss of *not* led to a marked increase in histone H2B ubiquitination at the *tim* and *Pdp1*ε gene loci (Bu et al., 2020). This effect was amplified by simultaneous knockdown of *Nipped-A* (the *Drosophila* ortholog of the human schizophrenia-associated gene TRAPP), the histone acetyltransferase component of the SAGA complex (Bu et al., 2020; Mahesh et al., 2020). *Nipped-A* knockdown similarly results in a lengthened circadian period (1–3 h across independent RNAi lines), reduced rhythm power, and decreased expression of *tim* and *Pdp1*ε, specifically through increased H2B ubiquitination at their promoter regions (Bu et al., 2020; Mahesh et al., 2020).

Genetic manipulation demonstrates that not and Nipped-A function together to regulate circadian timing. Overexpression of *not* can rescue the period-lengthening phenotype caused by *Nipped-A* deficiency, whereas double knockdown of *not* and *Nipped-A* synergistically exacerbates circadian defects (Bu et al., 2020). Although the mammalian *not* ortholog, *USP22*, has not yet been directly linked to clock regulation, the finding that H2B ubiquitination at mammalian clock gene loci was found to regulate clock gene transcription in mouse livers supports a conserved role for the SAGA DUBm in circadian rhythm control (Tamayo et al., 2015).

## 5 Ubiquitin pathways and their impact on health and disease

### 5.1 Disruption of circadian rhythms and UPS in human health

Disruptions in the circadian clock and UPS have been increasingly implicated in several disease states, particularly where proteostasis and time-dependent biological processes intersect. For instance, during sleep, the UPS facilitates the clearance of neurotoxic proteins like β-amyloid, and disruptions in circadian regulation can impair this clearance, contributing to disorders such as Alzheimer’s disease (Xie et al., 2013; Zee and Vitiello, 2009; Leng et al., 2019; La Spada and Ranum, 2010). Other neurodegenerative disorders including Parkinson’s, Huntington’s, and ALS have also been linked with UPS dysfunction, via contributing to the accumulation of toxic aggregates such as α-synuclein and huntingtin (Tseng et al., 2008; Dugger and Dickson, 2017; Venkatraman et al., 2004; Adori et al., 2005).

In cancer, circadian disruption–triggered by irregular light exposure or shift work–has been linked to increased tumor risk and progression, partly due to dysregulation in clock-controlled ubiquitin-mediated degradation of oncogenes and tumor suppressors such as p53 (Reszka and Zienolddiny, 2018; Fagiani et al., 2022). Similarly, in metabolic disorders like obesity and type 2 diabetes, E3 ligases (e.g., WWP1) and deubiquitinating enzymes (e.g., USP1, USP2, USP19, USP20) modulate insulin signaling and glucose homeostasis, processes that are governed by the circadian clock (Hirata et al., 2019; Yang et al., 2023). Furthermore, USP2 is rhythmically expressed across multiple tissues and is also induced by starvation, suggesting it integrates circadian and metabolic signals (Kita et al., 2002; Yan et al., 2008; Molusky et al., 2012b). It has been shown that liver USP2 contributes to the generation of a diurnal rhythm in glucose metabolism, linking UPS activity to circadian regulation of metabolic homeostasis (Molusky et al., 2012a).

## 6 Conclusion and future directions

The intricate relationship between the UPS and circadian rhythms is a fundamental axis of biological regulation. Ubiquitination and deubiquitination shape circadian timing by modulating the stability, activity, and localization of core clock proteins. E3 ubiquitin ligases often act with remarkable specificity, targeting substrates in a time- and compartment-dependent manner. In contrast, DUBs appear to be less selective, exhibiting regulatory roles across multiple clock components including PER1, CRY1, and BMAL1. Notably, these modifications do not act in isolation but are integrated within a broader network of post-translational modifications, amplifying the complexity of circadian control.

Despite significant advances, major gaps remain in our mechanistic understanding of the relationship between the UPS and circadian rhythms. The temporal dynamics and tissue-specific functions of many UPS components are still poorly defined. While rhythmic expressions of select E3 ligases and DUBs have been reported, their time-of-day-specific substrates, functional activities, and linkage-type preferences (e.g., K48 vs. K63) *in vivo* are largely unexplored. Addressing these questions will require time-resolved ubiquitin proteomics and tissue-specific *in vivo* loss-of-function studies across circadian timepoints to understand how the UPS both encodes and responds to circadian signals at the molecular level.

Finally, integrating circadian biology into the development of pharmacological modulators of UPS activity presents an exciting direction. For example, recent chemical screens have identified small molecules that bind to CRY proteins and modulate their ubiquitin-mediated degradation, offering a potential strategy for resetting of the clock (Hirota et al., 2012). More broadly, compounds targeting core circadian regulators such as REV-ERBs, RORs, CRYs, and CK1 have already shown potential to fine-tune clock-controlled physiology in preclinical models (Solt et al., 2012; Sulli et al., 2018; Chen et al., 2018c). Combining these with temporal targeting of UPS modulators could optimize the timing and efficacy of interventions, even outside of disease contexts, by aligning therapeutic activity with endogenous biological rhythms. As circadian and precision medicine continues to evolve, a more mechanistic understanding of UPS-circadian interplay will provide a foundation for rational design of time-based interventions.
